# proNGF/NGF mixtures induce gene expression changes in PC12 cells that neither singly produces

**DOI:** 10.1186/1471-2202-15-48

**Published:** 2014-04-08

**Authors:** Ivan Arisi, Mara D’Onofrio, Rossella Brandi, Francesca Malerba, Francesca Paoletti, Andrea Ennio Storti, Fulvio Florenzano, Luisa Fasulo, Antonino Cattaneo

**Affiliations:** 1 Genomics Facility, European Brain Research Institute (EBRI) “Rita Levi-Montalcini”, Via del Fosso di Fiorano, 64, 00143 Roma, Italy; 2 Istituto di Farmacologia Traslazionale, CNR, Via del Fosso del Cavaliere 100, 00133 Roma, Italy; 3 Neurotrophic Factors and Neurodegenerative Diseases Laboratory, European Brain Research Institute (EBRI) “Rita Levi-Montalcini”, Via del Fosso di Fiorano, 64, 00143 Roma, Italy; 4 European Brain Research Institute (EBRI) “Rita Levi-Montalcini”, Confocal Microscopy Unit, Via del Fosso di Fiorano, 64, 00143 Roma, Italy; 5 Scuola Normale Superiore, Piazza dei Cavalieri 7, 56126 Pisa, Italy

**Keywords:** PC12, NGF, proNGF, Neurotrophin, Early genes, Gene expression, Transcription, NGF/proNGF ratio

## Abstract

**Background:**

Growing evidence shows that, *in vivo,* the precursor of Nerve Growth Factor (NGF), proNGF, displays biological activities different from those of its mature NGF counterpart, mediated by distinct, and somewhat complementary, receptor binding properties. NGF and proNGF induce distinct transcriptional signatures in target cells, highlighting their different bioactivities. *In vivo,* proNGF and mature NGF coexist. It was proposed that the relative proNGF/NGF ratio is important for their biological outcomes, especially in pathological conditions, since proNGF, the principal form of NGF in Central Nervous System (CNS), is increased in Alzheimer’s disease brains. These observations raise a relevant question: does proNGF, in the presence of NGF, influence the NGF transcriptional response and viceversa? In order to understand the specific proNGF effect on NGF activity, depending on the relative proNGF/NGF concentration, we investigated whether proNGF affects the pattern of well-known NGF-regulated mRNAs.

**Results:**

To test any influence of proNGF on pure NGF expression fingerprinting, the expression level of a set of candidate genes was analysed by qReal-Time PCR in rat adrenal pheochromocytoma cell line PC12, treated with a mixture of NGF and proNGF recombinant proteins, in different stoichiometric ratios. These candidates were selected amongst a set of genes well-known as being rapidly induced by NGF treatment. We found that, when PC12 cells are treated with proNGF/NGF mixtures, a unique pattern of gene expression, which does not overlap with that deriving from treatment with either proNGF or NGF alone, is induced. The specific effect is also dependent on the stoichiometric composition of the mixture. The proNGF/NGF equimolar mixture seems to partially neutralize the specific effects of the proNGF or NGF individual treatments, showing a weaker overall response, compared to the individual contributions of NGF and proNGF alone.

**Conclusions:**

Using gene expression as a functional read-out, our data demonstrate that the relative availability of NGF and proNGF *in vivo* might modulate the biological outcome of these ligands.

## Background

NGF, the prototype member of the neurotrophin protein family, is translated as a pre-pro-protein, processed by furin protease in the trans-Golgi network or by extracellular proteases, to give rise to mature NGF [1]. Besides its suggested roles in the regulation of neurotrophin secretion [2] and as an intramolecular chaperone for the folding of mature NGF [3], the precursor proNGF was found to display independent biological activities [4-6], different from those of its mature NGF counterpart, mediated by distinct, and somewhat complementary, receptor binding properties. In particular, proNGF binding with higher affinity to p75NTR [6] is increased by the presence of sortilin, a specific receptor for pro-neurotrophins [7]. Our recent finding demonstrates that NGF and proNGF activate distinct transcriptional signatures in target cells, highlighting their different bioactivities [8]. However, the relevant question whether the concomitant presence of proNGF and NGF affects the NGF transcriptional response has not been addressed yet. The interest in such analysis derives from a number of observations, in both physiological and pathological conditions.

First, proNGF and NGF coexist *in vivo*[9]. During ageing, proNGF increases in rat cortex and hippocampus, whereas NGF gradually decreases in the cortex. Second, in pathological conditions, such as prodromal and, more significantly, end-stage Alzheimer’s Disease, the levels of proNGF in the brain increase [10-12]. These alterations are suggested to play a role in the reduced brain plasticity and in the cognitive decline observed during ageing and neurodegeneration [13-15]. The balance between proNGF and NGF levels and signalling in the brain is thus a key determinant for brain homeostasis, with its disruption possibly leading to neurodegeneration [16,17]. We have previously characterized the distinct properties of proNGF and NGF signalling by gene expression microarray in PC12 cells and identified two subsets of differentially expressed genes that could be ascribed to a “pure proNGF” and a “pure NGF” signalling, respectively [8]. This initial characterization of the transcriptional signature of proNGF in PC12 cells confirmed that the mature and the precursor NGF proteins are biologically different, showing a different transcriptional signature. In order to understand the proNGF specific modulatory effect on NGF induced transcriptional activity and to test any influence of proNGF on “pure NGF” expression fingerprinting, PC12 cells were treated with a mixture of both recombinant proteins, in different stoichiometric ratios. Thus, we analysed the expression level of candidate genes, mainly selected among well-known NGF-induced genes [8,18-20]. A specific “proNGF effect” on NGF-induced gene expression was identified. Interestingly, a group of differentially expressed genes (about 50% of the analysed genes) shows two opposite trends 30 minutes after the single NGF or proNGF treatment. Moreover, when both neurotrophins are added, the coexistence of proNGF and NGF induces a “novel effect, peculiar to the mixture”, not induced by the single ligand addition.

## Results

### Experimental design and analysis overview

**Figure 1:**
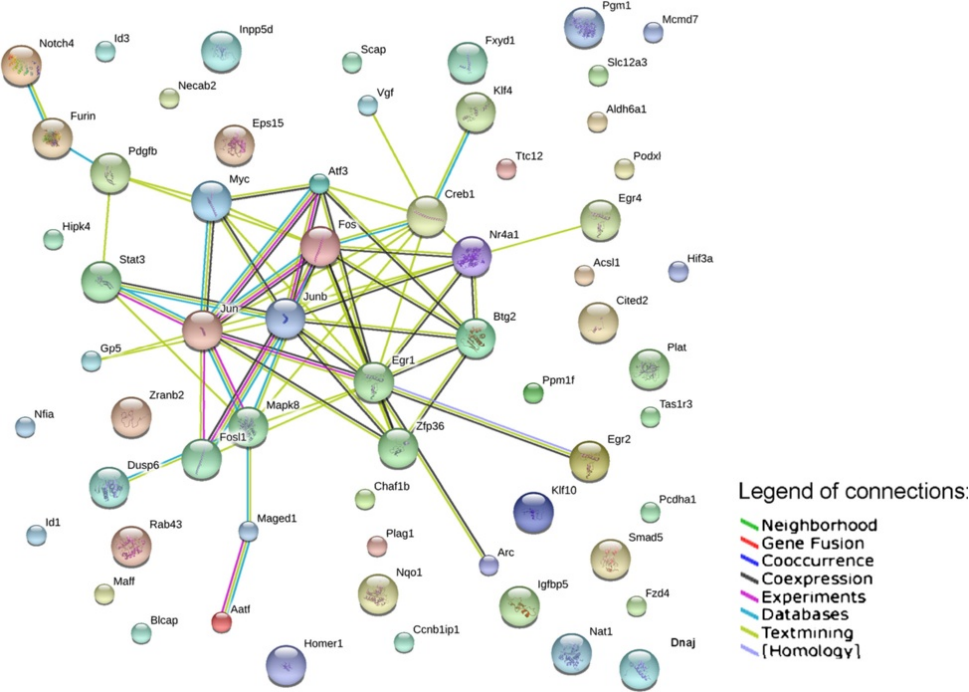
**Functional networks of analysed genes.** Functional connections among the analysed genes, according to the String protein-protein interaction database (http://string-db.org/), with High Confidence threshold parameter.

The expression of sixty-five genes was analysed, by qReal-Time PCR, in PC12 cells treated with only NGF, only proNGF or a mixture with both for different times (5, 15, 30 and 60 minutes), as schematized in Additional file 1. The set of genes was selected because of their well characterized response to NGF [18,8], or because they are involved in proNGF processing (directly, in the case of furin, or indirectly, in the case of tissue plasminogen activator) [2,21], or in modulating survival/apoptosis choices and differentiation programs. The functional interconnection between the selected genes was evaluated by means of the STRING database (http://string-db.org), a public repository of experimentally known and computationally predicted protein-protein interactions. The interaction network (Figure 1) shows that the genes are very sparsely connected and are thus independent, except for genes encoding for some transcription factors, early activated by NGF (*Jun, Fos, Creb, Myc* and few others [18,22-24]). Data analysis was carried out on the expression levels, after treatment with proNGF-only (proNGF/NGF 20/0 ng/ml or 200/0 ng/ml), NGF-only (proNGF/NGF 0/10 ng/ml) or proNGF/NGF mixtures (20/10 ng/ml, equimolar; 200/10 ng/ml; 40/10 ng/ml).

### Early effects of proNGF and NGF on gene expression

**Figure 2:**
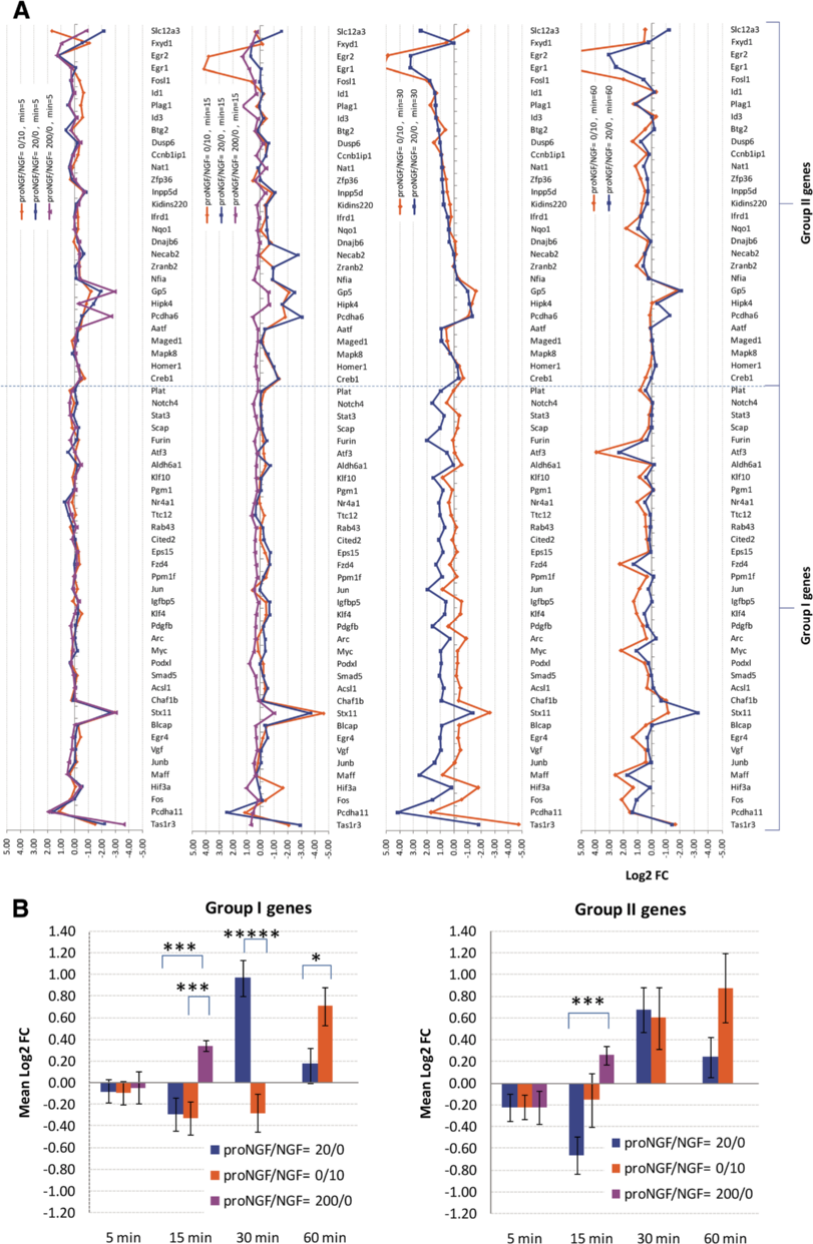
**Plots of Log2 Fold Change for analysed genes, following single neurotrophin treatments. (A)** Plots of Log2 Fold Change (FC) for the analysed genes, at 5, 15, 30, 60 minutes, with the two treatments: proNGF/NGF = 0/10 ng/ml and proNGF/NGF = 20/0 ng/ml. At 5 and 15 minutes, a third treatment with high proNGF is shown, proNGF/NGF = 200/0 ng/ml. The Group I genes show a stronger proNGF-mediated effect at 15 minutes with proNGF/NGF =200/0 and at 30 minutes with proNGF/NGF =20/0. **(B)** Average Log2 Fold Change ratio in the two groups of genes shown in **(A)**. The proNGF/NGF =200/0 and proNGF/NGF =20/0 treatments originate a similar response, with a significant increase in gene transcription at 15 and 30 minutes, respectively: this effect is even more significant for the genes of group I highlighted in Figure 2**A**. (*****) 1-tail T-test with unequal variance, p < 0.00001. (***) 1-tail T-test with unequal variance, p < 0.001. (*) 1-tail T-test with unequal variance, p < 0.05. Error bars are s.e.m.

We recently showed that proNGF and NGF activate largely distinct transcriptional programs after 60 minutes of incubation [8]: in this work we extend the investigation to shorter time intervals. We characterized the gene expression profile of the chosen subset of genes in PC12 cells treated with proNGF (20 ng/ml) or NGF (10 ng/ml) for 5, 15, 30 and 60 minutes. Genes were grouped on the basis of their differential responses to proNGF or NGF alone (Figure 2A). It is noteworthy that already at 5 minutes the modulation of a number of mRNAs can be observed. This suggests a possible regulation at the translational level.

We identified two groups of genes mainly responding to proNGF (group I) or NGF only (group II), as reported in Figure 2A, (average Log2 Fold change ratio). As for the latter group, data show that proNGF is much slower in the induction of some IEGs, such as *Fosl1, Egr1, Egr2*[20,25] induced by NGF very early on (15 minutes). ProNGF induces a modest increase of the expression of these mRNAs only at 60 minutes (Figure 2A). At this time point, our previous analysis [8] had shown that the induction of these genes is much stronger in response to NGF than to proNGF. On the other hand, group I displays, at 30 minutes, an up-regulated response to proNGF (20 ng/ml) compared to that to NGF (10 ng/ml), which is weakly down-regulated. Interestingly, the trend for this group of genes is inverted after 1 hour of treatment, when the response to proNGF is reduced, and NGF shows an up-regulatory effect on the same genes, even larger than that displayed by proNGF (Figure 2A). The histogram in Figure 2B summarizes the responses to NGF or proNGF and shows that, at 30 minutes, the group I genes display a large up-regulation by proNGF, and a weak down-regulation by NGF. The histogram also shows that higher doses of proNGF (200 ng/ml) anticipate the up-regulated response observed at 30 minutes for the group I genes, differentially modulated by proNGF, displaying an earlier activity response (15 minutes) compared to the lower proNGF dose. As for the group II genes, the time point of 15 minutes highlights an overall opposite action of NGF, compared to the higher dose of proNGF. Log2 Fold change of the pure proNGF or NGF responses at the different time points analysed is reported in Additional file 1 (the group I is highlighted in light blue).

### The equimolar proNGF/NGF mixture induces changes in PC12 cells that neither singly produces

**Figure 3:**
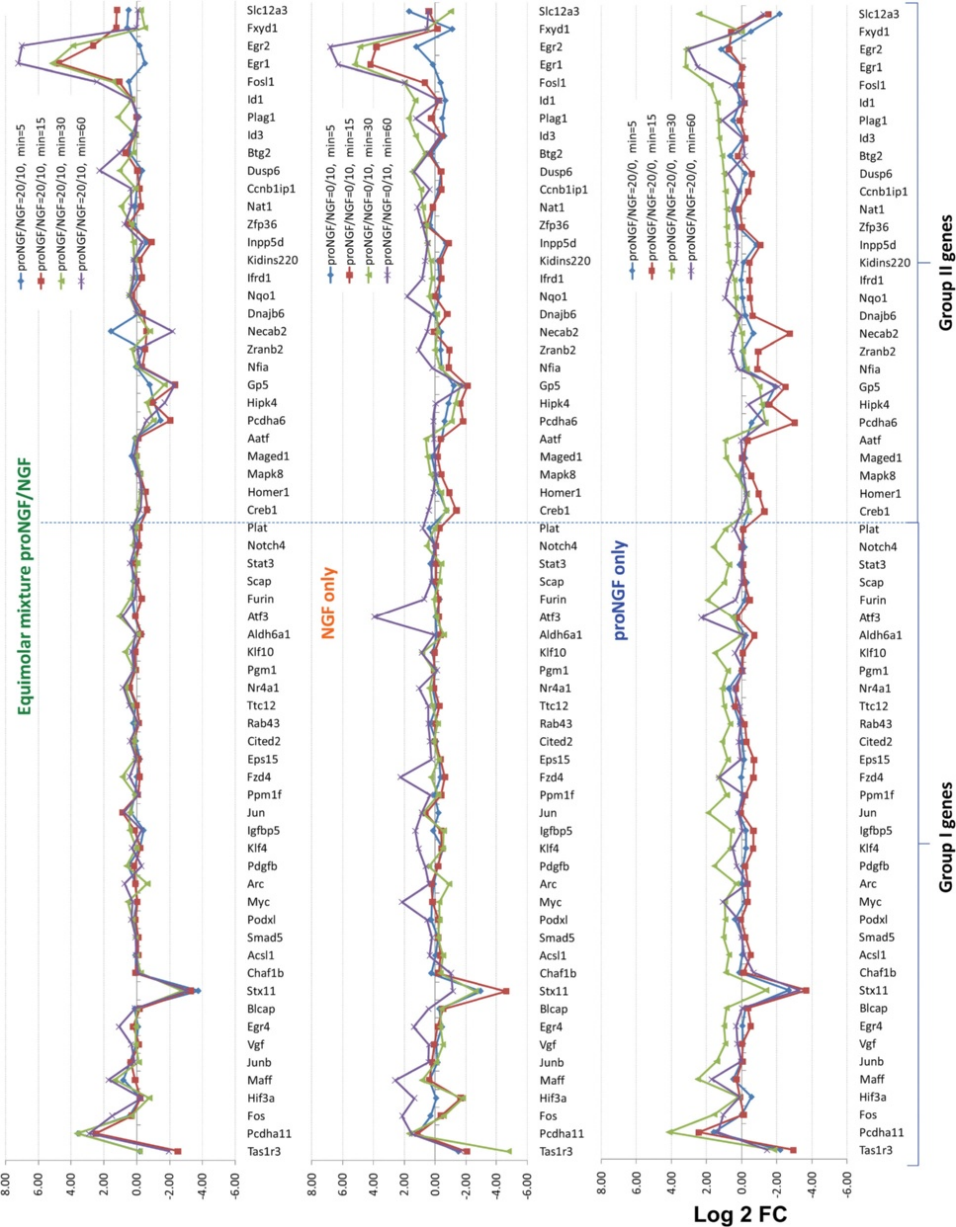
**Plots of Log2 Fold Change for analysed genes, following single neurotrophin and equimolar mixture treatments.** Plots of Log2 Fold Change for the 65 selected genes, with the treatments with NGF (proNGF/NGF 0/10 ng/ml), proNGF (proNGF/NGF 20/0 ng/ml) and the equimolar mixture of proNGF/NGF 20/10 ng/ml, each at 5,15,30 and 60 minutes.

**Figure 4:**
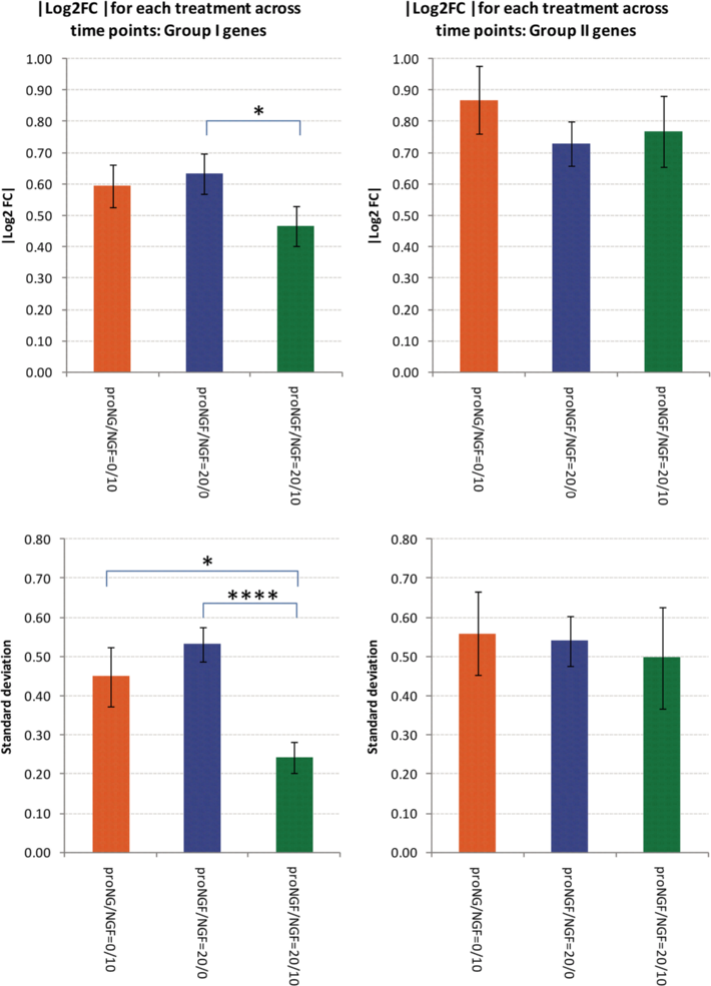
**Histograms of Log2 Fold Change for analysed genes, following single neurotrophin and equimolar mixture treatments.** (Upper panel) Average |Log2 Fold Change| (absolute value across time points) in the two groups of genes, with the treatments with NGF (proNGF/NGF 0/10 ng/ml), proNGF (proNGF/NGF 20/0 ng/ml) and the equimolar mixture of proNGF/NGF 20/10 ng/ml. (Lower panel) Average standard deviation (across time points) of |Log2 Fold Change| in the two groups of genes, with the treatments with NGF (proNGF/NGF 0/10 ng/ml), proNGF (proNGF/NGF 20/0 ng/ml) and the equimolar mixture of proNGF/NGF 20/10 ng/ml. The mixture shows a weaker response in Group I genes, as underlined also by a much smaller variability across time. (****) 1-tail T-test with unequal variance, p < 0.0001. (*) 1-tail T-test with unequal variance, p < 0.05. Error bars are s.e.m.

**Figure 5:**
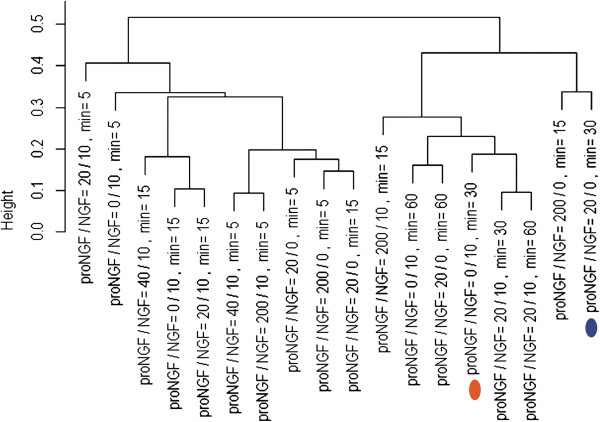
**Hierarchical clustering of samples.** Hierarchical clustering tree of samples, corresponding to the different experimental points (treatments and times). The proNGF/NGF concentrations are expressed in ng/ml. The tree shows the gene expression similarity between samples, by using the “non-centered Pearson” correlation, as a metric to compare the expression profiles. The y-axis indicates the distance between samples. The colours of highlighted treatments correspond to those displayed in Figure 4. This diagram shows that the degree of similarity between the NGF and proNGF responses at 30 minutes is low.

Regarding the question whether and how the coexistence of NGF and proNGF would influence the response of either ligand, we demonstrate that the proNGF/NGF equimolar mixture (20/10 ng/ml) shows coherent gene expression profiles at 5, 15, 30 and 60 minutes, without major differences at the different time-points analysed (Figure 3). The proNGF/NGF equimolar mixture seems to partially neutralize the specific effects of the proNGF or NGF individual treatments, showing a weaker overall response, compared to the individual contributions of NGF and proNGF alone (Figure 3). Figure 4 shows the average |Log2 Fold Change| and the average standard deviation of samples treated with NGF (proNGF/NGF 0/10 ng/ml), proNGF (proNGF/NGF 20/0 ng/ml) and the equimolar mixture (proNGF/NGF 20/10 ng/ml). The equimolar mixture shows a weaker response in Group I genes, as underlined also by a much smaller standard deviation, hence a reduced variability across time. Hierarchical clustering of samples in response to different proNGF/NGF treatments was computed (Figure 5). First, cluster analyses confirmed the earlier response to proNGF compared to the NGF one. The branching diagram represents a hierarchical tree of categories (different treatments) based on degree of similarity in the expression response. Different treatments showing similar expression response are closer in the tree; the distance is proportional to inter-samples dissimilarity. The chosen metric (“non centered Pearson” correlation) takes into account both the shape of profiles and the expression levels, compared to the commonly used “centered Pearson” correlation. The diagram in Figure 5 highlights the low degree of similarity between the NGF and proNGF responses at 30 minutes, whereas the NGF response is closest to the proNGF one at 60 minutes, with a high degree of similarity. Moreover, treatments with NGF, both pure and in mixtures, cluster together while proNGF is farther in the tree, supporting the first conclusion of a different gene activation pattern by proNGF compared to NGF.

### Time courses of responses

**Figure 6:**
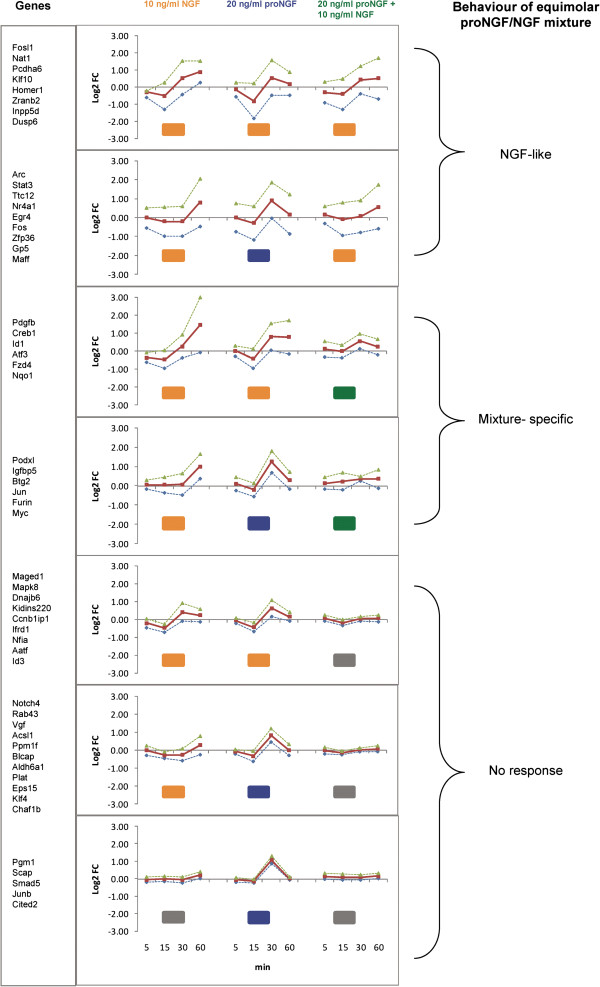
**Time course of gene expression profiles.** The figure shows the summary plots of expression profiles of selected genes, in three different experimental conditions, at 5, 15, 30 and 60 minutes after treatment. The expression level is the Log2 Fold Change ratio relative to the untreated condition. Each subplot is composed by three lines: the average expression value for the genes written on the left (red), average – standard deviation (dotted green), average + standard deviation (dotted blue). Every frame corresponds to a different cluster of genes and is composed by three plots: the response to 10 ng/ml NGF (left), the response to 20 ng/ml proNGF (center), the response to a mixture of 10 ng/ml NGF + 20 ng/ml proNGF (right). To evaluate the similarity between the responses to the three treatments the “centered Pearson” correlation coefficients (NGF, proNGF), (NGF, mixture), (proNGF, mixture) have been computed for each gene expression profile. The colours of the small rectangles below each plot indicate the similarity between the responses within each cluster, based on the correlation coefficients (where coefficients > 60% the profiles are considered similar): orange = NGF-like profile, blue = proNGF-like profile, green = mixture-like profile, grey = almost no response. The notes on the right summarize the response to the mixture of 10 ng/ml NGF + 20 ng/ml proNGF.

To evaluate how the responses to proNGF alone or to an equimolar mixture of NGF and proNGF differ from the NGF response, the similarity between the responses at different times were computed with a correlation coefficient (“centered Pearson”). The time-course of the gene expression profiles at 4 time points (5, 15, 30, 60 minutes) in 3 experimental conditions (10 ng/ml NGF, or 20 ng/ml proNGF or a mixture of both) was analysed and compared, as shown in Figure 6. In the graphical representation, each subplot corresponds to the average expression responses for a group of genes that shows a homogeneous response pattern. In PC12 cells exposed to a mixture of 10 ng/ml NGF plus 20 ng/ml proNGF (1:1 stoichiometry), response patterns are defined as “mixture-specific responses” peculiar to the mixture, and different from the profile observed after the addition of NGF or proNGF alone. A group of expression response patterns is identified, that mimics the trend observed after the single addition of NGF, defined as “NGF-like”. proNGF-like responses are not observed, upon mixture treatment with 1:1 ratio (Figure 6). Several examples, indicated as “no response” in Figure 6, are shown, after addition of the proNGF/NGF equimolar mixture. Such observation suggests a potential mutual antagonist role of proNGF and NGF in the equimolar range, such that each ligand cancels and counteracts the response to the other, confirming the overall result described in Figure 4, where the equimolar mixture of NGF and proNGF induces a reduced modulation for some of the analysed genes.

### Effects of increasing the proNGF concentration in the proNGF/NGF mixture

**Figure 7:**
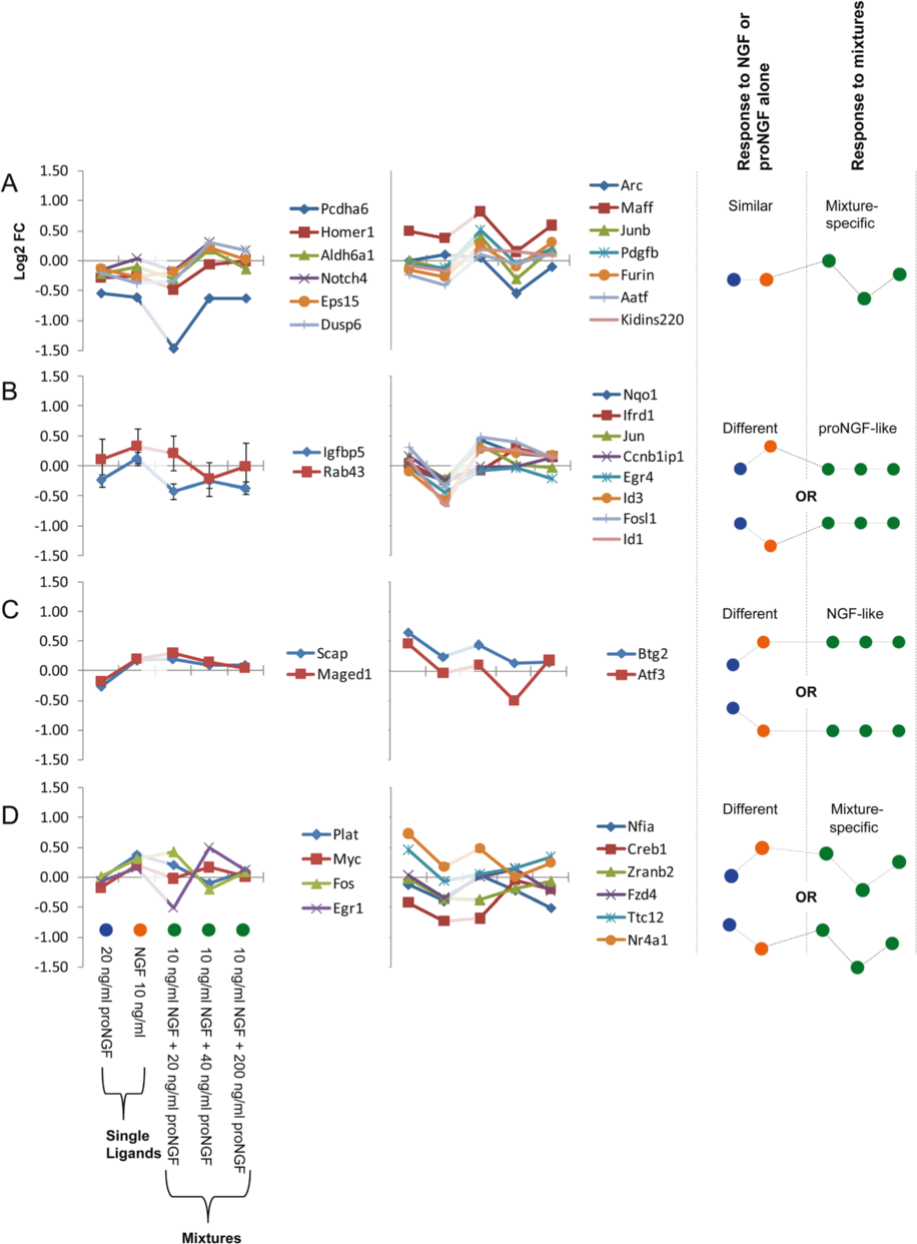
**Expression profiles of different proNGF/NGF mixtures at 5 minutes.** The figure shows the behaviour of genes in five different experimental conditions, 5 minutes after the treatments. Each single line plot is composed by 5 experimental points, respectively: 20 ng/ml proNGF, 10 ng/ml NGF, proNGF/NGF = 20/10 ng/ml, proNGF/NGF = 40/10 ng/ml, proNGF/NGF = 200/10 ng/ml. Expression profiles have been clustered based on two criteria: i) the responses to single NGF and proNGF ligands are similar or different ii) the shape of the response to mixtures and their similarity to the response to ligands alone. **A)** Response to NGF or proNGF alone: similar. Response to mixtures: variable depending on the proNGF/NGF ratio; left: first cluster of response profiles, right: second cluster of response profiles. **B)** Response to NGF or proNGF alone: different. Response to mixtures: closer to proNGF; left: response to NGF alone > proNGF, right: response to NGF alone < proNGF. **C)** Response to NGF or proNGF alone: different. Response to mixtures: closer to NGF; left: response to NGF alone > proNGF, right: response to NGF alone < proNGF. **D)** Response to NGF or proNGF alone: different. Response to mixtures: variable depending on the proNGF/NGF ratio; left: response to NGF alone > proNGF, right: response to NGF alone < proNGF. The notes and the prototype behaviours on the right summarize the prototypical responses to the single neurotrophins and to the mixtures. Colours of treatments (blue, orange, green) are the same as Figure 6.

**Figure 8:**
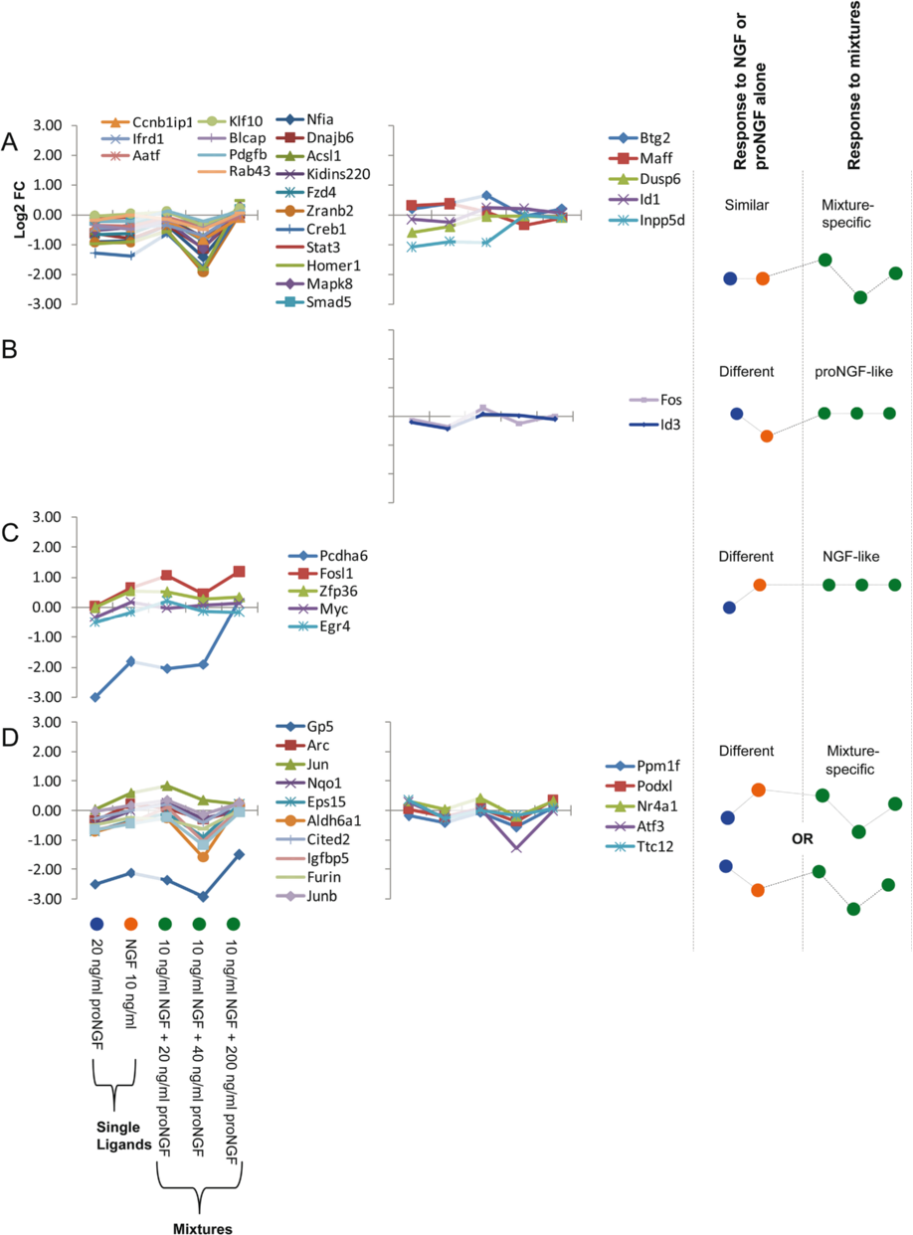
**Expression profiles of different proNGF/NGF mixtures at 15 minutes.** The figure shows the behaviour of genes in five different experimental conditions, 15 minutes after the treatments. Each single line plot is composed by 5 experimental points, respectively: 20 ng/ml proNGF, 10 ng/ml NGF, proNGF/NGF = 20/10 ng/ml, proNGF/NGF = 40/10 ng/ml, proNGF/NGF = 200/10 ng/ml. Expression profiles have been grouped based on two criteria: i) the responses to single NGF and proNGF ligands are similar or different ii) the shape of the response to mixtures and their similarity to the response to ligands alone. **A)** Response to NGF or proNGF alone: similar. Response to mixtures: variable depending on the proNGF/NGF ratio; left: first cluster of response profiles; right: second cluster of response profiles, with a saturation effect with proNGF/NGF increasing ratio. **B)** Response to NGF or proNGF alone: different. Response to mixtures: similar to proNGF. **C)** Response to NGF or proNGF alone: different. Response to mixtures: similar to NGF. **D)** Response to NGF or proNGF alone: different. Response to mixtures: variable depending on the proNGF/NGF ratio; left: response to NGF alone > proNGF; right: response to NGF alone < proNGF. The notes on the right summarize the prototypical responses to the single neurotrophins and to the mixtures. Colours of treatments (blue, orange, green) are the same as Figure 6.

We analysed the behaviour of individual genes, in response to treatments with NGF or proNGF alone (as a reference for comparison to pure NGF or pure proNGF responses) or with mixtures of NGF and proNGF, either at equal stoichiometry or with increasing amount of proNGF (20 ng/ml proNGF, 40 ng/ml proNGF, and 200 ng/ml proNGF plus 10 ng/ml NGF, corresponding respectively to stoichiometric ratios of 1:1, 2:1, and 10:1). The interest in evaluating the expression responses to increasing doses of proNGF derives from *in vivo* data, showing the coexistence of both proNGF and NGF [21,26] and the increased proNGF levels in pathological conditions [10,11]. Therefore, the modulatory effect of proNGF on genes known to be NGF-responders is an interesting and relevant issue, particularly at early time points (5 minutes, see Figure 7 and 15 minutes, see Figure 8), when limited proteolysis of proNGF has the chance to occur. Expression patterns for induced genes were clustered according to two parameters: i) response to individual NGF or proNGF ligands (are they similar or different?) and ii) response to the proNGF/NGF mixture (is it similar to the response to either ligand alone?).

At the 5 minutes time point, the following categories of expression patterns were identified (Figure 7):

A) Response to NGF or proNGF alone: similar. Response to mixtures: mixture-specific, depending on the proNGF/NGF ratio; two representative groups of response profiles were identified (A, left and right).

B) In the first group (B left), the response to NGF is higher than the response to proNGF alone, in the second (B right) it reverses. The response to the proNGF/NGF mixture is more similar to the response to proNGF alone (proNGF-like). C) Response to NGF or proNGF alone: different. Response to NGF alone: lower (C, left) or greater (C, right) than to proNGF. Response to mixtures: closer to NGF (NGF-like); this appears to be the least represented category. D) Response to NGF or proNGF alone: different. Response to NGF alone: lower (D, left) or greater (D, right) than to proNGF. Response to mixtures: mixture specific, depending on the proNGF/NGF ratio. A similar analysis was performed at 15 minutes, and groups of expression patterns were classified, according to the same criteria (Figure 8): A) Response to NGF or proNGF alone: similar. Response to mixtures: mixture-specific, depending on the proNGF/NGF ratio; two clusters of response profiles are shown, the second showing a saturation effect with proNGF/NGF increasing ratio. B) Response to NGF or proNGF alone: different. Response to proNGF/NGF mixtures: similar to proNGF. C) Response to NGF or proNGF alone: different. Response to mixtures: similar to NGF. D) Response to NGF or proNGF alone: different. Response to mixtures: mixture-specific, depending on the proNGF/NGF ratio. Compared to the 5 minutes data sample (Figure 7), two response patterns are not represented in the 15 minutes dataset (Figures 8B and C).

At 5 minutes (Figure 7), the responses to NGF or proNGF alone differ frequently; in those cases, the response to mixtures is variable, either similar to proNGF or NGF-responses, or peculiar to the proNGF/NGF mixture (mixture-specific). At 15 minutes (Figure 8), when the responses to NGF or proNGF alone differ, most frequently represented genes show a mixture-specific response, different from the NGF- or proNGF-responses. Moreover, increasing the amount of proNGF in the mixture (20 ng, 40 ng and 200 ng/ml) does not always produce a linear dose–response outcome in the expression pattern of the genes under consideration. Indeed, the response is often reverted, at the highest proNGF concentration (see Figure 8, cluster A left panel and cluster D).

**Figure 9:**
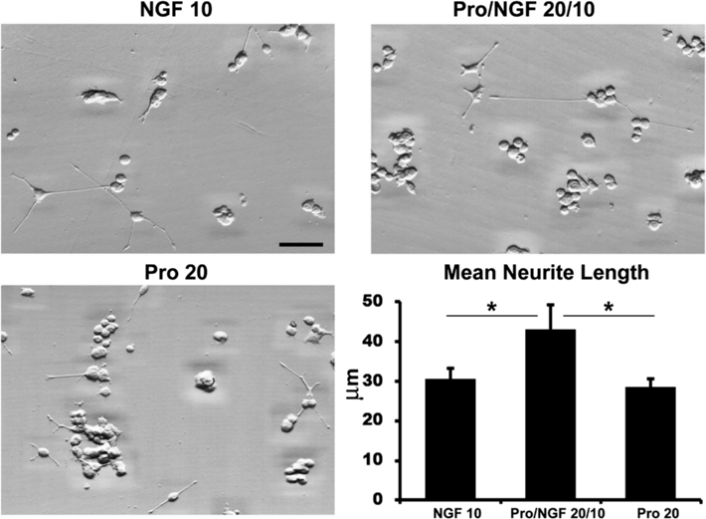
**Differentiation PC12 assay after 10 ng/ml NGF, 20/10 ng/ml proNGF/NGF, 20 ng/ml proNGF treatments.** DIC (Differential Interference Contrast) images show differentiated PC12 cells after 5 days of treatment. The graph displays the mean neurite length for each group. Values are mean + Standard Deviation (SD) of three independent experiments and statistically significant differences were calculated by unpaired-two tailed t-Student’s test (*p < 0.05 versus proNGF/NGF group). Note the increase in neurite length in the proNGF/NGF group compared to the NGF or proNGF alone treatments. Scale bar 40 μm.

To support the concept that NGF and proNGF mixture behaves differently from either NGF or proNGF alone, we analysed the morphological changes triggered by the addition of 10 ng/ml NGF, 20 ng/ml proNGF or an equimolar mixture of both molecules (proNGF/NGF 20/10 ng/ml). Differentiated PC12 were analysed on the basis of the neurite mean length for each type of treatment. Under our experimental conditions, neurite length assessment showed that, after 5 days of treatment, the proNGF/NGF 20/10 ng/ml group of genes exhibit a significant increase in the neurite mean length accounting for a 41% and 52% change (Figure 9), with respect to the 10 ng/ml NGF, or 20 ng/ml proNGF. The increase in the mean neurite length indicates a different response of the proNGF/NGF treated group compared to the single treatments.

## Discussion

The role of proNGF is controversial, and both neurotrophic and apoptotic activities have been reported for recombinant proNGF. It has been shown that proNGF can also be neurotrophic, regardless of mutations or tags, and no matter how it is purified or in which system it is expressed. However, although proNGF is neurotrophic for primary sympathetic neurons and for PC12 cells, it is reported to be apoptotic for unprimed PC12 cells [27]. Furthermore, proNGF has been reported to promote apoptosis via its interaction with p75NTR and sortilin receptor [7,28]. Both proNGF and mature NGF exhibited neurotrophic activity on PC12 cells, while the pro-domain itself promotes cell death [29]. NGF and proNGF co-exist *in vivo*[9]. One might therefore expect that the proNGF/NGF mixture modulates the downstream effect of either neurotrophin form, in ways depending on their ratio, and distinct from NGF and proNGF alone. However, it is not known whether and how the concomitant presence of proNGF affects cell responses to NGF.

Our data refer to the PC12 cells system and to mRNAs expression read-out as a response signature. This experimental system was chosen, together with the gene expression read-out, as a well defined system, for investigating the complex question of the functional consequences of signalling by mixtures of NGF/proNGF, in comparison to signalling by pure proNGF or pure NGF. We expect that proNGF/NGF mixtures might modulate the downstream responses, depending on their ratio, in ways distinct from those affected by NGF or proNGF alone, also in different systems than PC12 cells. It remains however to be ascertained whether and how the distinct signalling behaviours of the NGF/proNGF mixture, here demonstrated, also apply to neuronal cells. Thus, we have analysed by qReal-Time PCR the expression of a set of genes, in PC12 cells treated for short times with proNGF, NGF or with a mixture of the two proteins. The emphasis on short times was to minimize the chance of processing of proNGF by cellular proteases in the culture medium, which is known to occur at times longer than a few hours [8].

From our results, we conclude that there are a significant number of cases where proNGF has a prevalent effect over NGF. This effect is time dependent, as well as dependent on the relative concentration of the two proteins. Distinct proNGF-specific effects on NGF-induced gene expressions were identified. It should be pointed out that we do not imply to put forward a functional interpretation of the gene expression changes observed. Rather, we take the gene expression patterns as a fingerprinting read-out of possible actions of proNGF/NGF mixtures, as compared to those regulated by NGF or proNGF alone. Most of the selected genes are essentially functionally unrelated (Figure 1) and any correlated behaviour, observed in the PC12 cellular model, could be attributed to the specific treatment.

Taking into account single additions, among the selected panel of genes, about 50% of Group I shows an opposite trend in the single NGF or proNGF treatments at 30 minutes. Higher doses of proNGF anticipate at 15 minutes the proNGF up-regulated response, observed at 30 minutes for that group of genes. This suggests there may be a proNGF-induced dose-dependent effect on early signalling. Hierarchical clustering of samples highlights this low degree of similarity between the NGF and proNGF responses at 30 minutes. When NGF and proNGF were added simultaneously, the effect of the mixture on gene expression was often different from the effect of the single ligands added separately. Although some NGF-like responses were observed for the mixture (indicating proNGF being neutral), in many cases the mixture behaved differently. In particular, in the case of an equimolar mixture of proNGF and NGF, synergic, additive or mutually antagonizing effects were observed for different mRNAs. We can therefore identify a non-linear “proNGF effect” interfering with the NGF-induced gene expression.

Increased proNGF levels are observed during human ageing and pathological conditions [11,12]. This prompted us to analyse the effect of increasing proNGF concentration in the proNGF/NGF mixture. Different patterns of expression responses were obtained. At 5 minutes, the responses to NGF or proNGF alone are often different; in those cases, the response to mixtures shows a variable pattern, either similar to proNGF-response or to NGF-response or peculiar to the mixture, whereas at 15 minutes, the most represented group of genes shows a mixture-specific response. Moreover, the response is not always linear with the increasing amount of proNGF, being often reverted at the highest concentration. We conclude that the proNGF/NGF mixture shows a mixture-specific signature, that at these early time points (5 and 15 minutes) is not necessarily only transcriptional, but may involve translational regulation [30]. This aspect warrants to be further investigated. Our data support the hypothesis that the relative concentration of NGF and proNGF might be of importance for the biological outcome of the two proteins, in physiological and/or pathological conditions and suggests that the proNGF/NGF balance is a sensitive point of regulation in the homeostasis of the system. We have recently described a transgenic mouse model expressing a form of proNGF resistant to furin cleavage [17], showing that different expression levels of NGF and proNGF produce neurodegeneration phenotypes of different strength in transgenic lines derived from different founders [17]. The relative NGF and proNGF availability has been reported to regulate innervation density in postnatal Superior Cervical Ganglion (SCG) neurons: sub-saturating concentrations of proNGF and NGF act synergically to promote neurite growth, whereas the additive effect is not observed at saturating concentration. Conversely, lack of synergic effect was observed in trigeminal neurons, highlighting that subsets of NGF-responsive neurons have distinctive responses to NGF and proNGF [31]. Our data show that the mixture of proNGF and NGF acts differently, depending on the proNGF/NGF ratio, highlighting that conflict, synergism and/or cancellation occur when both NGF and proNGF downstream pathways are similarly activated through different receptors. The biochemical site of this conflict or synergism is currently unknown, and most likely involves interactions at the level of the signalling pathways, starting from the receptor(s) level and determining distinct downstream functional consequences. In any case, the interactions between the NGF and proNGF signalling pathways, when concomitantly activated, can be described as if the proNGF/NGF mixture functionally behaves as a “new ligand”, distinct from either NGF or proNGF alone.

## Conclusions

The regulation of proNGF and NGF relative concentrations might be a highly relevant parameter governing the final fate of a target cell, between differentiation, maintenance of a differentiated phenotype, survival or death. Different proNGF/NGF mixtures exert a fine tuning of distinct receptors binding and thus their differential signalling. In this complex scenario, we have discovered, under well defined experimental conditions, that the proNGF/NGF ratio induces a specific response, up-regulating or down-regulating some genes, differently than the individual proNGF or NGF ligands alone. Thus, we show that the proNGF/NGF mixture induces gene expression changes in PC12 cells that neither singly produces. This opens the possibility that *in vivo* a similar functional interaction between NGF and proNGF signalling may be operative.

The main conclusion is that proNGF is clearly modulating NGF gene expression and novel effects, peculiar to the mixture, are detected. The data support the idea that the relative proNGF/NGF concentration might specifically contribute to different biological activities, both in physiological and pathological conditions. Further studies will include blocking NGF receptors with specific antibodies to dissect the pathways involved and the receptor and signalling specificity, downstream of the proNGF/NGF mixture.

## Methods

### PC12 cell culture and treatment

Rat pheochromocytoma PC12 cells [32] (PC12 SB subclone) were maintained with RPMI 1640 Medium (Invitrogen) and grown as monolayer cultures on Falcon dishes, supplemented with 10% Horse Serum (Invitrogen) and 5% Foetal Calf Serum (Invitrogen), in a humidified atmosphere at 37°C and 5% CO_2_. PC12 cells were plated at a concentration of 10_6_/dish (100 mm plates, BD Falcon) and kept in culture for 12 hours or 5 days for imaging procedures. Cells were divided in four groups: PC12 treated with 10 ng/ml NGF, 20 ng/ml proNGF, with different proNGF/NGF mixtures or untreated PC12 cells (zero concentration of either ligand) (control group), following different times of incubation (5, 15, 30 and 60 minutes). NGF/ProNGF concentrations ranged from 1, 2 and 10 fold proNGF (in stoichiometrical terms), according to [8]. Two biological replicates were used for each time point and treatment. For imaging procedures cells received a medium change every two days, and were treated every day with the same molecules concentrations as described above. An additional group, which did not receive treatment (control group), was used for cell survival and neurite length assessment.

### Imaging and cellular analysis

For imaging procedures plated cells were monitored every day and images documentation were taken on an inverted microscope (TiE; Nikon, Japan) equipped with phase contrast objectives, with a cooled CCD camera (Clara; Andor) and Niss Elements imaging software (Nikon). Image processing and analysis were performed by using Imaris Suite 7.4® (Bitplane A.G., Zurich, Switzerland) or Image J 1.4 (http://rsbweb.nih.gov/ij/) softwares.

For assessment of neurite length, five fields (640 × 480 micron) randomly distributed, and derived from three replicates of each experimental group, were taken with a 20× objective after 5 days of incubation. Due to the high propensity of differentiated cells to form bidimensional and tridimensional contact clusters, a manual strategy for neurite length was adopted. For assessment of neurite length, images were opened with Imaris and neurite were traced with the line structure measurement tool. Only neurites, which were clearly identifiable, were considered for analysis. On the basis of pilot analyses on untreated cells, we decided to consider only neurite which protruded at least for 5 μm from the cell body.

For production of figures, bright field images of cells were taken with an objective 10× under a confocal laser scanning microscope (Leica SP5, Leica Microsystems, Wetzlar, Germany) equipped with a transmitted light detector for Differential Interference Contrast (DIC; Nomarski) acquisitions. Images were adjusted for brightness and contrast, inhomogeneities of the brightness across the image were corrected with Adobe Photoshop high-pass filter and contrast of cellular edges was enhanced with ImageJ CLAHE (Contrast Limited Adaptive Histogram Equalization; http://rsbweb.nih.gov/ij/plugins/clahe/index.html) filter plugin (block size, 89; histogram bins, 256; maximum slope, 2). Final figures were assembled by using Adobe Photoshop 6 and Adobe Illustrator 10.

### RNA extraction

RNA was isolated from PC12 cells treated with NGF, proNGF or different neurotrophin composition, following different incubation times, as described above. PC12 cell cultures were scraped and lysed with Trizol (Invitrogen) and DNAse treated by Qiagen columns. RNA quantity was determined on a NanoDrop UV–VIS. Only samples with an absorbance ratio of 1.8 < OD_260_/OD_280_ < 2.0 were processed further. Each sample was then quality checked for integrity using the Agilent BioAnalyzer 2100 (Agilent G2938C, RNA 6000 nano kit).

### Real-time qRT-PCR

The expression data of a set of mRNA candidates was evaluated by qRT-PCR, using the two-step Applied Biosystem 7900HT Micro Fluidic Card protocol. To analyse the variation in gene expression levels of various commonly used endogenous controls, a TaqMan® LowDensity Endogenous Control Panel was used (Applied Biosystems, Foster City, CA). Sixty-five genes and 3 housekeeping genes were analysed (68 genes). For quantification of gene expression changes, the Ct method was used, to calculate relative fold changes normalized against the housekeeping genes: ribosomal *RNA 18S*, TATA binding protein (*Tbp*) and glyceraldehyde 3- phosphate dehydrogenase (*Gapdh*).

### Data analysis

The Group I genes (Figures 2, 3 and 4) were selected using specific criteria. In summary, we chose genes that clearly show a different response between the proNGF-only and NGF-only treatments, particularly at 30 minutes. First, for each gene at time t, we calculated the difference between the Log2 fold change with the proNGF-only and NGF-only treatments. Calling these differences DeltaFC (gene_i, t) where i = (1…65) and t = (5,15,30,60) minutes. Each one of the eventually selected 36 genes satisfies all the following rules at the same time:

a) MAX(DeltaFC(gene_i, 5), DeltaFC(gene_i, 15),DeltaFC(gene_i, 30), DeltaFC(gene_i, 60) = DeltaFC(gene_i, 30)

b) DeltaFC(gene_i, 30) > 0.5

c) DeltaFC(gene_i, 60) < 0.0 OR DeltaFC(gene_i, 60) < DeltaFC(gene_i, 30)

d) |DeltaFC(gene_i, 5)| < |DeltaFC(gene_i, 30)|

e) |DeltaFC(gene_i, 15)| < |DeltaFC(gene_i, 30)|

Significant differences highlighted in Figures 2B and 4 were obtained by 1-tail T-test with unequal variance between samples. Hierarchical clustering analysis in Figure 5 was obtained using R-Bioconductor. Temporal profiles in Figure 6 were obtained in Microsoft Excel by clustering genes with a “centered Pearson” correlation coefficient > 0.6. Concentration profiles in Figures 7 and 8 were obtained in Microsoft Excel and MultiExperiment Viewer ver. 4.6 [33] based on their shapes, in particular taking into account the difference between response to single neurotrophins and to the NGF/proNGF mixtures. The network in Figure 1 was built using StringDB (http://string-db.org), a protein-protein interaction database.

## Abbreviations

NGF: Nerve growth factor; proNGF: proNerve growth factor; CNS: Central nervous system; SCG: Superior cervical ganglion; CLAHE: Contrast limited adaptive histogram equalization; DIC: Differential interference contrast; Tbp: TATA binding protein; Gapd: Glyceraldehyde 3- phosphate dehydrogenase; FC: Fold change; SD: Standard deviation.

## Competing interests

The authors have no commercial association that might create a competing interest in connection with the submitted manuscript.

## Authors’ contributions

MD, IA, RB, AC: conception and design; MD, FP, AES, IA, RB, FF, FM, LF, acquisition of data; MD, FP, IA, RB, FM, FF, AES, LF, AC: analysis and interpretation of data; MD, IA, RB, FP, FM, LF, AC: drafting and revising the manuscript. All authors read and approved the final manuscript.

## Supplementary Material

Additional file 1 **Log2 Fold Change ratios for the analysed selected genes.** Table of Log2 Fold Change ratio for the analysed 65 selected genes, at 5, 15, 30, 60 minutes, with the three treatments, where proNGF/NGF = P/N in ng/ml: NGF-only with P/N = 0/10, proNGF-only with P/N = 20/0 or P/N = 200/0. The 36 genes with a stronger proNGF-mediated effect at 30 minutes are highlighted in light blue. Colour scale corresponds to Fold Change values. Click here for file
