# Supplementary material for: proNGF/NGF mixtures induce gene expression changes in PC12 cells that neither singly produces
Source: BMC Neurosci. 2014 Apr 8;15:48. doi: 10.1186/1471-2202-15-48 (PMC4098786; doi:10.1186/1471-2202-15-48)
Supplement: Additional file 1 — Log2 Fold Change ratios for the analysed selected genes. Table of Log2 Fold Change ratio for the analysed 65 selected genes, at 5, 15, 30, 60 minutes, with the three treatments, where proNGF/NGF = P/N in ng/ml: NGF-only with P/N = 0/10, proNGF-only with P/N = 20/0 or P/N = 200/0. The 36 genes with a stronger proNGF-mediated effect at 30 minutes are highlighted in light blue. Colour scale corresponds to Fold Change values. [file 1471-2202-15-48-S1.pdf]

Additional File 1. Log2 fold change ratios for the analyzed selected genes.

Log2 FC

-1.0 +1-0

scale:

Symbol	Name	proNGF/NGF= 0/10 , min=5	proNGF/NGF= 20/0 , min=5	proNGF/NGF= 200/0 , min=5	proNGF/NGF= 0/10 , min=15	proNGF/NGF= 20/0 , min=15	proNGF/NGF= 200/0 , min=15	proNGF/NGF= 0/10 , min=30	proNGF/NGF= 20/0 , min=30	proNGF/NGF= 0/10 , min=60	proNGF/NGF= 20/0 , min=60
Tas1r3	taste receptor, type 1, member 3	-1.49	-2.18	-3.59	-2.08	-2.94	0.68	-4.78	-1.83	-1.68	-1.44
Pcdha11	protocadherin alpha 11	1.15	1.61	1.95	1.14	2.44	0.57	1.67	4.14	1.52	1.36
Fos	FBJ osteosarcoma oncogene	0.30	0.01	0.22	-0.37	-0.12	0.13	-0.54	1.57	2.13	1.05
Hif3a	hypoxia inducible factor 3, alpha subunit	-0.06	-0.54	-0.42	-1.66	0.10	1.04	-1.78	0.18	1.33	0.12
Maff	v-maf musculoaponeurotic fibrosarcoma oncogene homolog F (avian)	0.37	0.48	0.55	0.37	0.31	0.34	0.85	2.52	2.59	1.71
Junb	jun B proto-oncogene	-0.15	-0.01	0.41	0.19	-0.04	0.49	-0.07	1.42	0.42	0.03
Vgf	VGF nerve growth factor inducible	-0.05	-0.05	0.19	0.05	-0.03	0.23	-0.49	0.96	0.40	0.26
Egr4	early growth response 4	-0.43	-0.04	0.16	-0.18	-0.51	0.33	-0.33	1.01	1.37	0.31
Blcap	bladder cancer associated protein homolog (human)	-0.26	-0.20	0.01	-0.51	-0.36	0.37	-0.40	0.88	0.42	-0.02
Stx11	syntaxin 11	-2.96	-2.68	-3.01	-4.65	-3.68	-1.01	-2.65	-1.38	-1.16	-3.29
Chaf1b	chromatin assembly factor 1, subunit B (p60)	0.24	0.14	-0.03	-0.24	-0.11	0.09	-0.33	0.91	-1.02	-0.67
Acsl1	acyl-CoA synthetase long-chain family member 1	-0.02	-0.05	-0.02	-0.34	-0.51	0.29	-0.50	0.74	0.34	-0.13
Smad5	SMAD family member 5	-0.18	-0.03	0.07	-0.22	-0.21	0.38	-0.17	1.05	0.20	0.02
Podxl	podocalyxin-like	0.26	0.37	0.29	-0.22	0.04	0.82	-0.28	0.94	0.52	0.22
Myc	myelocytomatosis oncogene	0.20	-0.18	0.14	0.16	-0.34	0.49	-0.25	0.96	2.15	1.07
Arc	activity-regulated cytoskeleton-associated protein	0.10	-0.01	0.15	0.22	-0.33	0.34	-0.90	0.30	0.39	-0.32
Pdgfb	platelet-derived growth factor beta polypeptide (simian sarcoma viral (v-sis) oncogene homolog)	-0.13	-0.08	0.29	-0.21	-0.20	0.37	0.41	1.58	0.62	0.29
Klf4	Kruppel-like factor 4 (gut)	-0.51	-0.26	0.01	-0.44	-0.66	0.31	-0.49	0.67	1.08	0.51
Igfbp5	insulin-like growth factor binding protein 5	0.13	-0.24	-0.30	-0.42	-0.67	0.14	-0.55	0.58	1.29	-0.03
Jun	Jun oncogene	-0.21	0.16	0.10	0.60	0.05	0.49	0.82	1.93	0.86	0.22
Ppm1f	protein phosphatase 1F (PP2C domain containing)	0.05	-0.03	0.04	-0.42	-0.19	0.22	-0.20	0.86	0.33	-0.15
Fzd4	frizzled homolog 4 (Drosophila)	-0.35	0.02	-0.16	-0.65	-0.67	0.30	0.27	1.32	2.25	1.28
Eps15	epidermal growth factor receptor pathway substrate 15	-0.28	-0.13	-0.17	-0.35	-0.71	0.40	-0.22	0.80	0.22	0.06
Cited2	Cbp/p300-interacting transactivator, with Glu/Asp-rich carboxy-terminal domain, 2	-0.04	-0.02	0.21	0.01	-0.27	0.42	0.11	1.11	0.34	0.17
Rab43	RAB43, member RAS oncogene family	0.33	0.11	-0.14	-0.02	-0.18	0.32	-0.17	0.67	0.42	0.07
Ttc12	tetratricopeptide repeat domain 12	-0.07	0.45	0.23	-0.30	0.36	0.64	0.19	1.01	0.46	0.09
Nr4a1	nuclear receptor subfamily 4, group A, member 1	0.17	0.72	0.51	0.05	0.31	0.43	0.38	1.10	1.05	0.46
Pgm1	phosphoglucomutase 1	-0.02	0.04	-0.04	0.06	-0.03	0.22	0.10	0.81	-0.12	-0.10
Klf10	Kruppel-like factor 10	0.16	-0.03	-0.01	0.03	-0.06	0.31	0.85	1.53	0.86	0.39
Aldh6a1	aldehyde dehydrogenase 6 family, member A1	-0.10	-0.22	-0.46	-0.39	-0.72	0.33	-0.56	0.03	0.04	-0.17
Atf3	activating transcription factor 3	-0.04	0.46	0.00	-0.25	0.26	0.21	-0.07	0.50	3.94	2.30
Furin	furin (paired basic amino acid cleaving enzyme)	-0.28	-0.16	0.32	-0.23	-0.47	0.45	0.08	1.97	0.72	0.35
Scap	SREBF chaperone	0.17	-0.27	-0.08	-0.06	-0.12	0.17	-0.26	1.01	0.23	-0.01
Stat3	signal transducer and activator of transcription 3	0.30	0.09	0.33	-0.08	-0.10	0.35	-0.39	0.72	0.19	0.00
Notch4	Notch homolog 4 (Drosophila)	0.03	-0.15	0.38	-0.05	0.01	0.53	0.55	1.59	-0.03	-0.06
Plat	plasminogen activator, tissue	0.37	-0.02	0.21	-0.28	-0.09	0.19	0.05	0.95	0.81	0.42
Creb1	cAMP responsive element binding protein 1	-0.73	-0.43	-0.45	-1.38	-1.30	0.21	-0.69	-0.37	0.42	0.01
Homer1	homer homolog 1 (Drosophila)	-0.26	-0.29	-0.23	-0.92	-0.97	0.36	-0.37	-0.26	0.10	-0.29
Mapk8	mitogen-activated protein kinase 8	-0.01	0.13	-0.05	-0.41	-0.55	0.33	0.27	0.26	-0.02	-0.11
Maged1	melanoma antigen, family D, 1	0.20	-0.18	-0.03	-0.16	-0.04	0.20	0.50	0.91	0.01	-0.05
Aatf	apoptosis antagonizing transcription factor	-0.42	-0.24	-0.18	-0.38	-0.35	0.21	0.59	0.94	0.07	0.05
Pcdha6	protocadherin alpha 6	-0.61	-0.54	-2.68	-1.83	-3.03	0.57	-1.06	-1.36	0.13	-1.28
Hipk4	homeodomain interacting protein kinase 4	-0.86	-1.38	-0.24	-1.64	-1.57	-0.64	-1.31	-1.15	-0.03	-0.38
Gp5	glycoprotein V (platelet)	-1.20	-1.91	-2.93	-2.11	-2.51	-0.57	-1.63	-1.01	-1.82	-2.09
Nfia	nuclear factor I/A	-0.40	-0.13	-0.33	-0.89	-0.91	0.47	-0.36	-0.27	0.18	0.22
Zranb2	zinc finger, RAN-binding domain containing 2	-0.36	-0.03	-0.29	-0.94	-0.95	0.19	0.03	-0.06	1.06	0.58
Necab2	N-terminal EF-hand calcium binding protein 2	-0.38	-0.67	-0.33	0.07	-2.75	0.17	-0.13	-0.01	0.50	0.47
Dnajb6	DnaJ (Hsp40) homolog, subfamily B, member 6	0.07	-0.22	-0.33	-0.80	-0.62	0.06	-0.11	0.32	0.24	0.05
Nqo1	NAD(P)H dehydrogenase, quinone 1	-0.25	-0.05	0.02	-0.01	-0.48	0.21	0.39	0.35	1.81	0.94
Ifrd1	interferon-related developmental regulator 1	-0.25	0.03	-0.02	-0.42	-0.45	0.09	0.22	0.44	0.84	0.73
Kidins220	kinase D-interacting substrate 220	-0.19	-0.10	-0.26	-0.36	-0.45	0.26	0.27	0.75	0.65	0.33
Inpp5d	inositol polyphosphate-5-phosphatase D	-0.70	-0.83	-0.68	-0.89	-1.08	-0.37	0.52	0.80	0.48	0.25
Zfp36	zinc finger protein 36	0.33	0.20	-0.01	0.55	-0.02	0.39	0.58	0.87	0.80	0.29
Nat1	N-acetyltransferase 1	0.15	0.41	0.34	0.14	0.15	-0.41	0.79	0.87	1.16	0.55
Ccnb1ip1	cyclin B1 interacting protein 1	-0.24	0.15	0.13	-0.42	-0.38	0.29	0.92	0.93	0.34	0.17
Dusp6	dual specificity phosphatase 6	-0.38	-0.21	-0.41	-0.39	-0.59	-0.19	1.48	1.01	1.34	0.76
Btg2	BTG family, member 2	0.24	0.65	0.30	0.38	0.21	0.41	0.64	1.12	0.46	-0.18
Id3	inhibitor of DNA binding 3	-0.58	-0.10	-0.14	-0.44	-0.21	0.19	1.26	1.30	-0.31	-0.03
Plag1	pleiomorphic adenoma gene 1	-0.45	0.47	0.43	0.23	0.10	1.32	1.71	1.33	1.26	1.11
Id1	inhibitor of DNA binding 1	-0.65	0.10	0.03	-0.24	-0.16	0.31	1.29	1.38	-0.30	-0.18
Fosl1	fos-like antigen 1	-0.35	0.31	0.25	0.64	0.02	0.45	1.98	1.77	1.99	0.57
Egr1	early growth response 1	0.15	-0.07	0.24	4.17	-0.04	0.84	5.20	3.21	6.29	2.53
Egr2	early growth response 2	1.22	1.19	1.30	3.80	0.72	1.33	4.87	3.19	6.81	3.05
Fxyd1	FXYD domain-containing ion transport regulator 1	-1.10	-0.52	0.98	-0.16	0.59	0.60	0.57	n.d.	0.52	0.22
Slc12a3	solute carrier family 12 (sodium/chloride transporters), member 3	1.68	-2.15	-0.87	0.39	-1.53	0.27	-1.01	2.43	0.45	-1.23

Additional File 1. Log2 Fold Change ratio for the analyzed selected genes, at 5, 15, 30, 60 minutes, with the three treatments, where proNGF/NGF=P/N in ng/ml: NGF-only with P/N=0/10, proNGF-only with P/N=20/0 or P/N=200/0. The 36 genes with a stronger proNGF-mediated effect at 30 minutes are highlighted in light blue. Color scale corresponds to fold change values.
